# Multistage preclinical evaluation of a retrievable 3 T MR-resonant inferior vena cava filter and snare loop for MRI-guided interventions

**DOI:** 10.1186/s42155-026-00770-z

**Published:** 2026-09-14

**Authors:** Denis Gholami Bajestani, C. Martin Reich, Jan Birkigt, Christina Mulik, Johannes Kauffold, Haukur Lindberg Sigmarsson, Lisa Wahl, Michael Schmid, Senta Schauer, Thies Jochimsen, Bernhard Sattler, Osama Sabri, Erwin Immel, Andreas Melzer

**Affiliations:** 1 Innovation Center Computer Assisted Surgery, Faculty of Medicine, Leipzig University, Leipzig, 04103 Germany; 2 Clinic for Ruminants and Swine, Faculty of Veterinary Medicine, Leipzig University, Leipzig, 04103 Germany; 3 EPflex Feinwerktechnik GmbH, Dettingen an der Erms, 72581 Germany; 4 Department of Nuclear Medicine, Leipzig University Hospital, Leipzig, 04103 Germany; 5 Institute for Medical Science and Technology, University of Dundee, Dundee, DD1 4HN UK

**Keywords:** MRI-guided intervention, Inferior vena cava filter, Interventional MRI, MR-resonant device, Snare loop, Endovascular intervention, MRI-guided retrieval

## Abstract

**Background:**

Fluoroscopy-guided inferior vena cava (IVC) filter implantation and retrieval involve ionizing radiation and iodinated contrast agents. MRI-guided interventions may provide a radiation-free alternative with direct visualization of filter position and hook accessibility. This study evaluated the feasibility of MRI-guided implantation and snare loop (SNL)-assisted retrieval of a dedicated MR-resonant IVC filter at 3 T.

**Methods:**

MR-resonant devices were assessed in a three-stage protocol. Device visibility was investigated in a saline phantom using balanced steady-state free precession (bSSFP) imaging at 3 T with the IVC filter oriented parallel to the main magnetic field B_0_ and assessed at different longitudinal-axis rotations and flip angles. MRI-guided IVC filter implantation was performed in a polymer-based vessel phantom. Finally, MRI-guided IVC filter implantation and SNL-assisted retrieval were performed in porcine models (*n* = 5).

**Results:**

MR-resonant signal enhancement enabled reliable visualization of the IVC filter in phantom and in vivo experiments. Eight filters were successfully implanted in five animals, and SNL-assisted retrieval followed by re-implantation was achieved in all retrieval attempts (3/3). Quantitative signal analysis of the saline phantom experiments demonstrated significant effects of flip angle (*p* < 0.001), rotation angle (*p* < 0.001), and filter diameter (*p* = 0.032) on normalized signal intensity, with the strongest signal enhancement observed at a flip angle of 10°.

**Conclusions:**

MRI-guided implantation and SNL-assisted retrieval of an MR-resonant IVC filter at 3 T demonstrated feasibility within a multistage preclinical workflow. Phantom and in vivo experiments showed reliable visualization and procedural guidance, supporting further development toward clinically applicable MRI-guided caval interventions.

**Graphical Abstract:**

**Figure:**
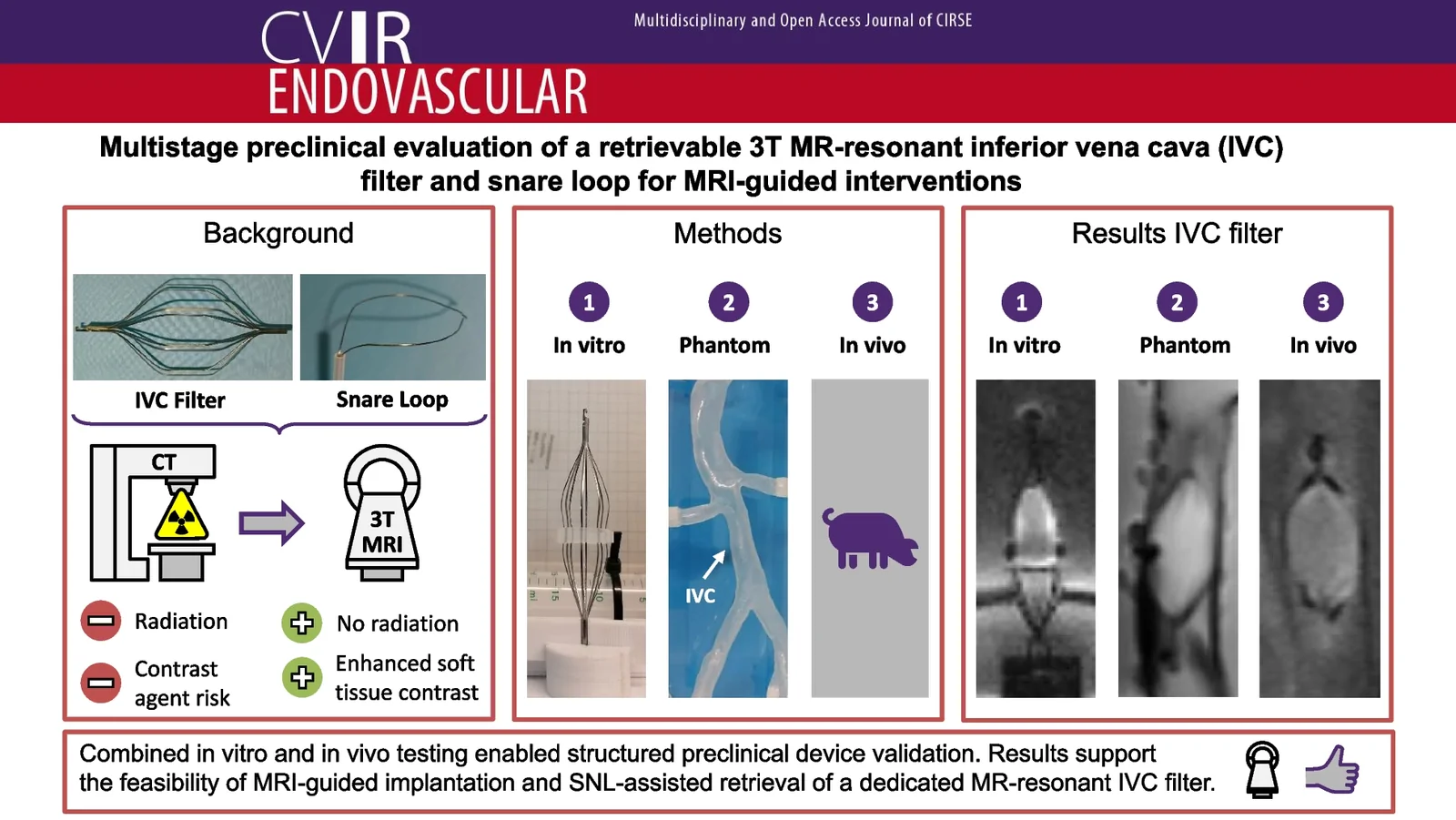

**Supplementary Information:**

The online version contains supplementary material available at https://doi.org/10.1186/s42155-026-00770-z.

## Background

MRI has emerged as a promising guidance modality for minimally invasive cardiovascular interventions by enabling radiation-free imaging, excellent soft tissue contrast, and simultaneous visualization of anatomy, devices, and physiological processes [1]. However, routine clinical implementation remains limited due to restricted availability of MR-compatible devices, challenges in real-time imaging, and the lack of standardized procedural workflows [2]. Inferior vena cava (IVC) filter implantation and retrieval are established minimally invasive procedures for prevention of venous thromboembolism in patients with contraindications to anticoagulation or recurrent embolic events [3, 4]. These interventions are typically performed under fluoroscopic guidance, exposing patients and staff to ionizing radiation and requiring iodinated contrast agents [5–7]. Further, retrieval procedures may become technically challenging in cases of prolonged implantation time, particularly when fibrin deposition or thrombus formation obscures the IVC filter hook and prevents capture with standard snare techniques [8, 9]. MRI-guided alternatives may reduce these limitations while enabling direct visualization of filter position, thrombus burden, vessel anatomy, and hook accessibility, potentially enabling image-based retrieval planning and MR-guided filter removal within the same session [10–12]. Therefore, the aim of this study was to develop and evaluate a MR-resonant IVC filter and a dedicated snare loop (SNL) system for MRI-guided implantation and retrieval at 3 T following a standardized workflow protocol.

## Materials and methods

### Study design

This study followed a three-stage preclinical evaluation framework comprising device visibility analysis (Stage I), phantom-based MRI-guided interventions (Stage II), and in vivo MRI-guided implantation and retrieval of MR resonant IVC filters in porcine models (Stage III). These stages addressed essential requirements for MRI-guided endovascular procedures, including visualization, workflow integration, and feasibility under physiological conditions.

### MR-resonant IVC filter and snare loop design

**Fig. 1:**
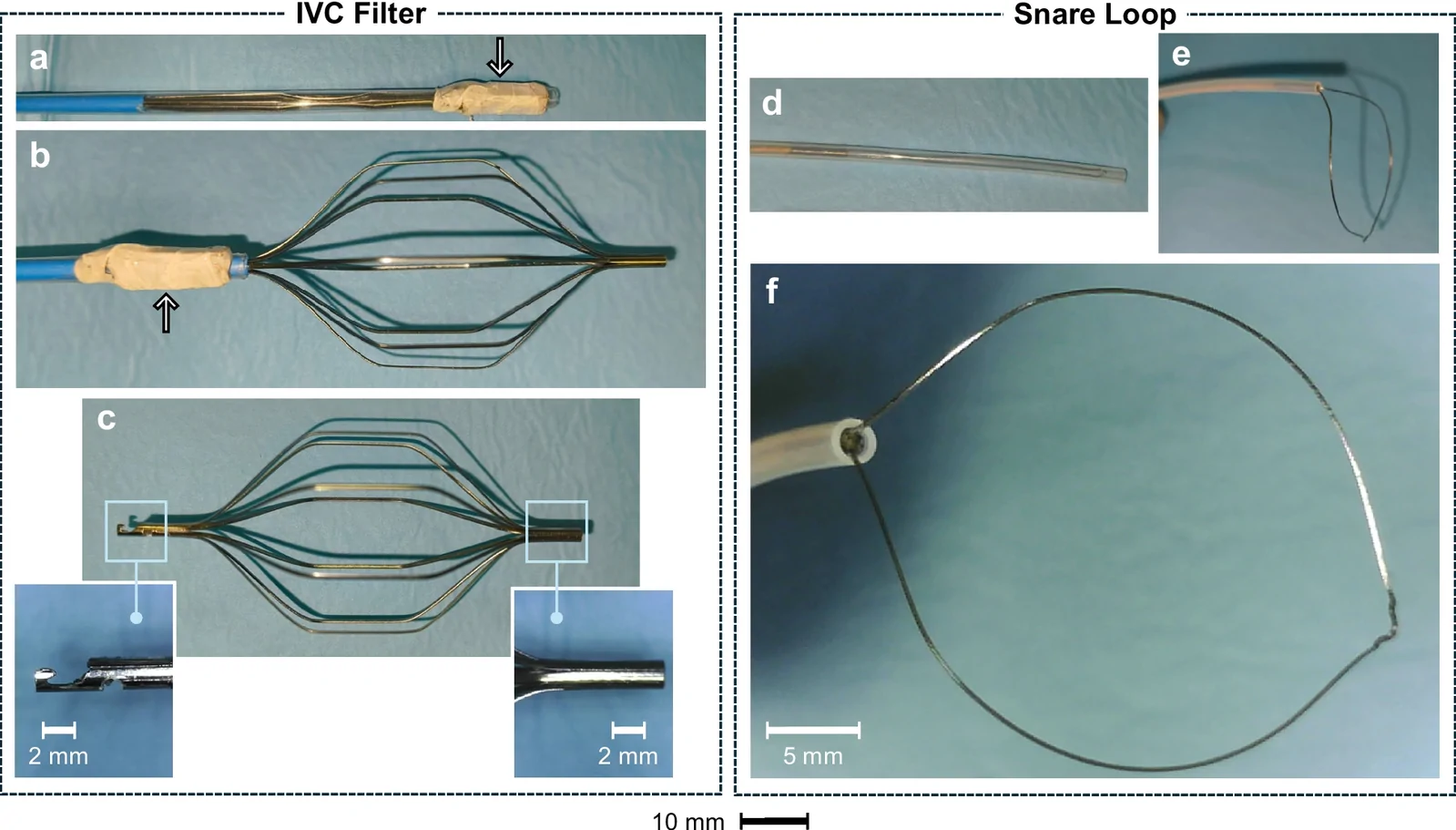
Inferior vena cava (IVC) filter and snare loop (SNL) designed for MRI-guided interventions. For catheter-guided delivery of the IVC filter, a custom-built guiding catheter equipped with a semi-active MR-resonant marker was used, whereas SNL–assisted retrieval was performed using a catheter with passive iron-oxide markers. **a** IVC filter positioned inside the semi-actively marked delivery catheter; the MR-resonant saddle coil marker is indicated by the white arrow. **b** IVC filter deployed from the delivery system; MR-resonant saddle coil marker indicated by the white arrow. **c** IVC filter with integrated resonant circuit design. The left inset shows a magnified view of the retrieval hook, while the right inset depicts the cranial-facing end of the IVC filter. **d** MR-resonant SNL integrated into a dedicated catheter-based delivery system with passively marked MR Conditional shaft. For retrieval, the system was used in combination with an outer retrieval catheter. **e** SNL deployed from the catheter. **f** Magnified view of the SNL

MRI-guided interventions were performed using self-expanding nitinol-based IVC filters and snare loops (Fig. 1a, b). Both devices were designed as MR-resonant structures tuned to the proton Larmor frequency at 3 T (128 MHz) [13]. Resonance was achieved using electrically insulated loop-antenna designs with integrated capacitance based on previously described concepts for resonant interventional devices [10, 12, 14]. The devices were manufactured from laser-cut nitinol tubing and thermomechanically expanded to final diameters of 30 mm (IVC filter) and 20 mm (SNL). Device development was performed at the Innovation Center Computer Assisted Surgery (Leipzig, Germany), while laser cutting and thermomechanical forming were performed by MeKo (Sarstedt, Germany). The design-integrated resonant inductor–capacitor (LC) circuit required electrically separated strut components to prevent short-circuiting between resonant elements. Mechanical integrity was maintained by medical-grade adhesive junctions at the proximal and distal filter ends and at the distal connector element of the SNL. Post-processing, adhesive assembly, and SNL handle integration were performed by Epflex Feinwerktechnik (Dettingen, Germany).

### MRI-guided interventional workflow and imaging setup

An MRI-guided workflow for catheter-based IVC filter implantation and retrieval was adapted from standard fluoroscopy-guided caval interventions [15]. Static balanced steady-state free precession (bSSFP) imaging (TrueFISP) was used for device visibility assessment, whereas real-time cine bSSFP sequences enabled device tracking during phantom and in vivo interventions. Across all stages, imaging protocols (Table 1) were iteratively adapted to support transfer from controlled bench testing to phantom and in vivo MRI-guided interventions.

**Table 1:** MRI sequence parameters used for staged evaluation of MR visibility and MRI-guided IVC filter implantation and retrieval using a snare loop on a 3 T Biograph mMR system (Siemens Healthineers) with customized TrueFISP imaging

Study context	TR^a^ (ms/frame)	TE (ms)	ST (mm)	FA (°)	BW (Hz/px)	Matrix	Pixel (mm^2^)	FoV (mm^2^)
Stage I. a: MR visibility assessment of resonant IVC filter (rotation and flip-angle series)	428.83	2.22	3	10, 20, 40	560	208 × 208	1.50 × 1.50	310 × 310
Stage I. b: MR visibility assessment of snare loop (sagittal and transverse orientation)	428.83	2.04	10	40	560	208 × 208	1.44 × 1.44	300 × 300
Stage II: In vitro phantom-based IVC filter implantation	394.12	2.04	5	40	560	208 × 208	1.44 × 1.44	300 × 300
Stage III: In vivo MRI testing	283.60	1.36	4.5	33	850	256 × 256	1.23 × 1.23	316 × 316

All sequences used GRAPPA acceleration (factor 2)

*Abbreviations*: *TR* temporal resolution, *TE* echo time, *ST* slice thickness, *FA* flip angle, *BW* bandwidth, *Pixel* pixel size, *FoV* field of view

^a^TR value corresponds to frame duration per cine phase, not sequence RF repetition time

### Stage I: device visibility assessment

The suitability of the developed MR-compatible interventional devices for MRI-guided procedures was first evaluated regarding MR visibility and resonant imaging behavior on a 3 T MRI system (Biograph mMR, Siemens Healthineers, Erlangen, Germany). For device visibility assessment, the IVC filter and SNL were immersed in a saline phantom (0.9% NaCl solution) and evaluated without their respective delivery systems (ESM-1). Static bSSFP (TrueFISP) acquisitions were used to characterize resonant signal behavior and overall image appearance.

To quantify MR-resonant behavior, signal intensity profiles were extracted along line profiles across the IVC filter and SNL. For IVC filter experiments, imaging was performed with the filter oriented parallel to the main magnetic field B_0_, corresponding to the expected anatomical orientation within the inferior vena cava. Measurements were acquired at different flip angles (10°, 20°, and 40°) and after rotation of the IVC filter around its longitudinal axis (22.5°, 45°, 67.5°, and 90°). A single MR-resonant IVC filter was evaluated at two constrained diameters (13 mm and 20 mm), representing different implantation states (ESM-1). For SNL experiments, the fully expanded loop was primarily oriented parallel to the axial imaging plane, reflecting its intended clinical application for IVC filter retrieval, with caudally oriented IVC filter hook. Measurements were performed at a flip angle of 40°, corresponding to the intended application flip angle for MRI-guided interventions and in vitro phantom studies. Additional MR images were acquired with orthogonal loop orientation (sagittal plane) to further assess the orientation-dependent resonance behavior observed in preliminary experiments.

Quantitative signal analysis was performed using a custom Python-based evaluation tool based on ASTM F2119 criteria (see ESM-2 for details).

### Stage II: in vitro phantom-based IVC filter implantation

**Fig. 2:**
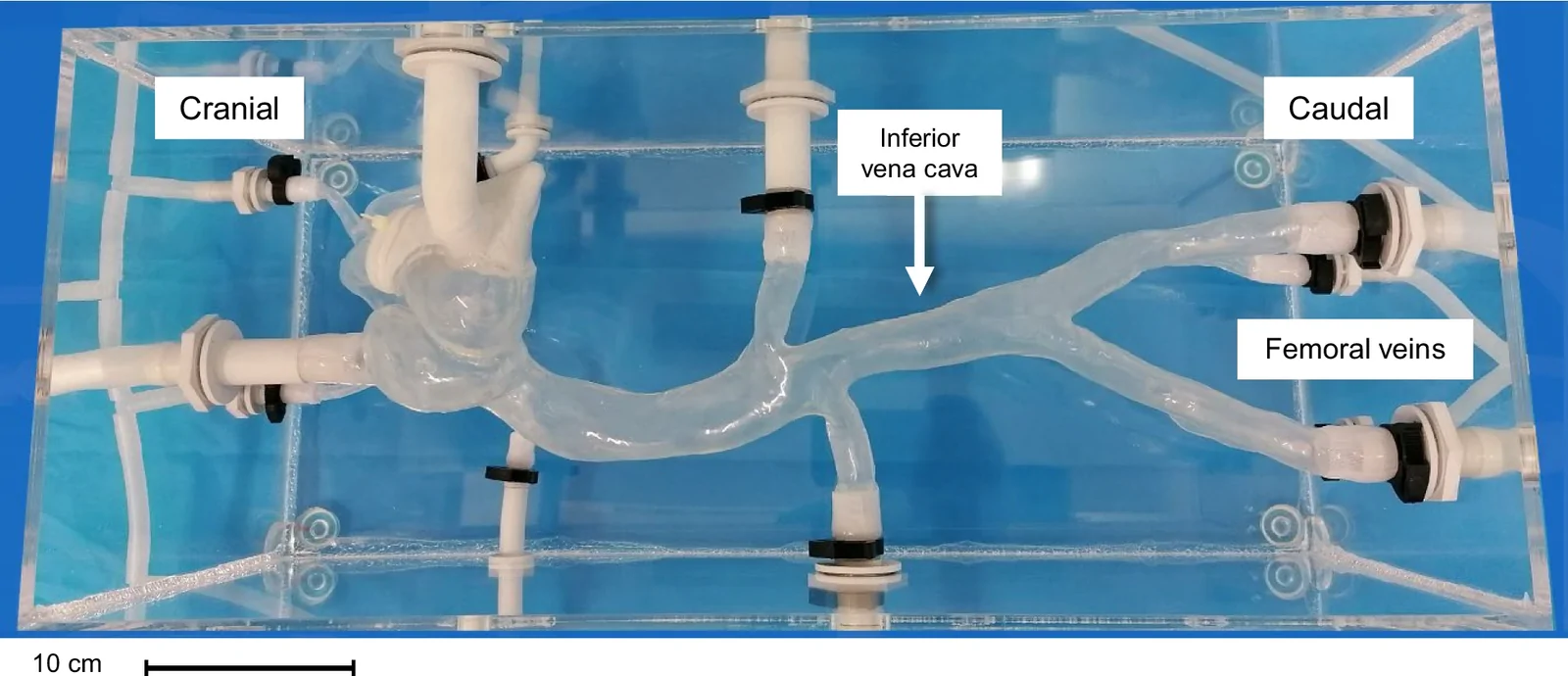
Top view of the scan-data-based silicone vessel phantom used for testing MRI-guided catheter-based inferior vena cava filter intervention. The vessel system extends from the femoral veins through the inferior vena cava, where the vena cava filter is implanted, to the right heart and pulmonary arteries

MRI-guided IVC filter implantation was tested using a polymer-based in vitro phantom (Fig. 2). The phantom was produced according to the previously published manufacturing protocol [16] based on CT data (59 y/o female) from the female Visible Human dataset (U.S. National Library of Medicine). Physiological pulsatile flow was simulated using a saline–glycerol mixture and a centrifugal pump system. Detailed phantom specifications are provided in ESM-3.1.

MRI-guided IVC filter implantation was simulated using the vascular phantom to assess procedural feasibility with MR-compatible devices. After target plane planning, the delivery system was advanced over an MR Conditional guidewire and positioned using the integrated MR-resonant marker. The IVC filter was subsequently advanced through the delivery system and deployed by pullback of the delivery sheath and advancement of a dedicated fiberglass pusher rod. Femoral access was established under ultrasound guidance outside the scanner room. For an initial qualitative assessment of thrombus visibility and interaction with the deployed filter under MRI guidance, an in vitro thrombus prepared from coagulated porcine blood obtained from a local abattoir was introduced via the deployment catheter and flushed through the vessel phantom toward the deployed IVC filter. Detailed instrumentation specifications are provided in ESM-3.2.

### Stage III: in vivo MRI testing

Five female pigs (*Sus scrofa domesticus*; mean body weight, 47.4 kg; range, 34–58 kg) underwent MRI-guided caval interventions as part of a larger ethically approved animal study evaluating MRI-guided cardiovascular interventions [17]. The operators had prior catheter-based interventional experience and practiced filter implantation and retrieval in the in vitro vessel phantom before the in vivo experiments, but had no prior clinical experience with IVC filter procedures. Animal preparation and physiological monitoring followed a previously established protocol [18]. All interventions were performed using the established MRI-guided workflow and the MR-compatible devices described above. The IVC filter was deployed via femoral access in an infrarenal position with the retrieval hook oriented caudally and was subsequently retrieved via the same access route. After deployment, the IVC filter was retrieved under MRI guidance using an SNL-based sheath retrieval technique. Detailed procedural information, including interventionist positioning during device manipulation, and ARRIVE statements are provided in ESM-4.

### Statistical analysis

An additive ordinary least-squares model with type II ANOVA was used to evaluate the main effects of filter diameter, rotation angle, and flip angle on normalized signal intensity. Interaction effects were not evaluated due to the absence of replicate measurements per experimental condition; *p* < 0.05 was considered significant, and partial eta squared (*ηp*^2^) was reported as effect size.

## Results

### Stage I: MR visibility of resonant IVC filter and snare loop

**Fig. 3:**
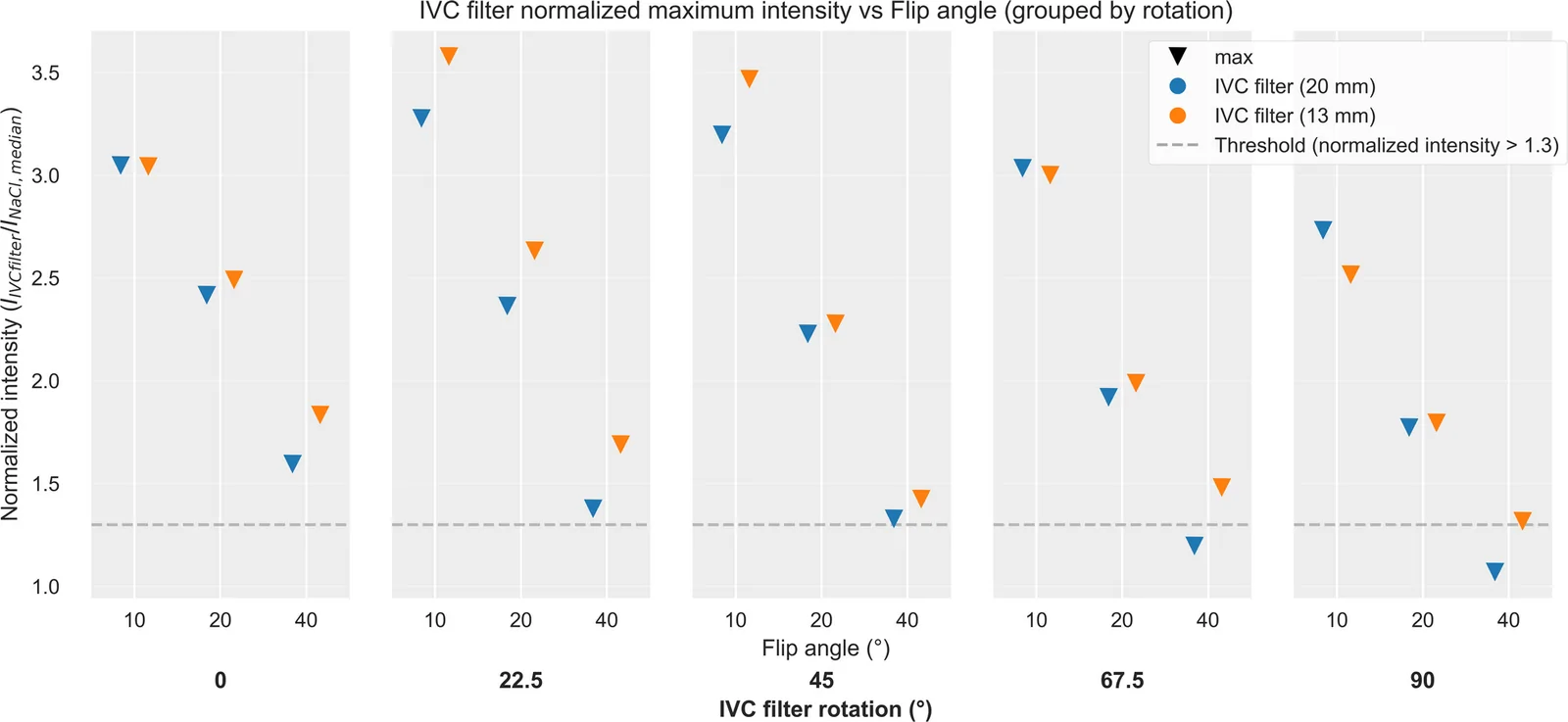
Normalized MR signal intensity maxima of a resonant IVC filter measured at different device rotations relative to the main magnetic field (B₀) and varying flip angles (10°, 20°, and 40°). Five grouped panels represent filter rotations between 0° and 90°. Results are shown for two diameter configurations of the same IVC filter (20 mm, blue; 13 mm, orange). Downward-pointing triangles indicate the maximum normalized signal intensity for each parameter combination (*I*_normalized_ = *I*_IVC filter_/*I*_NaCl median_). The dashed line indicates the predefined detection threshold (normalized intensity > 1.3). Only two configurations remained below the threshold, both involving the 20 mm filter at a flip angle of 40° and rotation angles of 45° and 90°

Normalized signal intensity analysis of the IVC filter demonstrated a dependence on flip angle across all rotation configurations (Fig. 3). For both investigated diameter configurations (13 mm and 20 mm), the highest normalized signal maxima were observed at a flip angle of 10°. Normalized signal maxima exceeding the predefined threshold (*I*_normalized_ > 1.3) were observed for most rotation and flip angle combinations. A factorial ANOVA revealed significant effects of all investigated attributes: diameter (F (1,22) = 5.22, *p* = 0.032, *ηp*^2^ = 0.19), rotation angle (F (4,22) = 15.13, *p* < 0.001, *ηp*^2^ = 0.73), and flip angle (F (2,22) = 275.71, *p* < 0.001, *ηp*^2^ = 0.96). Among the investigated factors, flip angle showed the largest effect size, followed by rotation angle, whereas filter diameter exhibited only a moderate influence on normalized signal intensity. Representative example images are provided in Figure ESM-5.1.

For the SNL, MR-resonant signal behavior was evaluated in two orientations at a flip angle of 40°. In the expected in vivo retrieval orientation (loop plane parallel to the transverse imaging plane), no signal enhancement exceeding the predefined threshold (*I*_normalized_ > 1.3) was observed, although MR signal visibility within the loop area remained preserved (see ESM-5.2 and ESM-5.3). In contrast, sagittal loop orientation resulted in resonant signal enhancement exceeding the threshold criterion (see ESM-5.4).

### Imaging workflow for MRI-guided IVC filter placement and retrieval

**Fig. 4:**
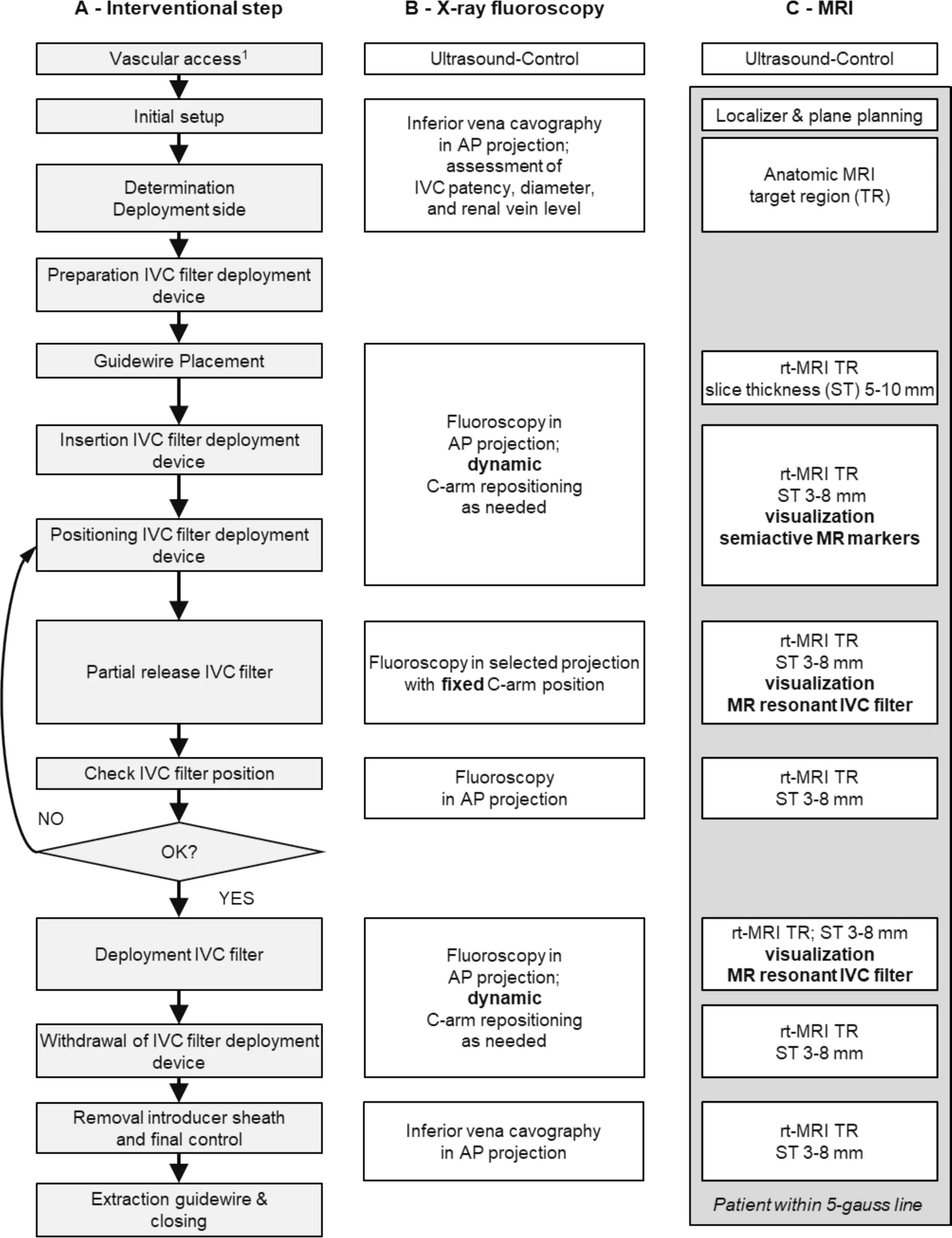
Flow diagram of catheter-based IVC filter implantation (**A**), the standard imaging workflow for fluoroscopy-guided IVC filter implantation (**B**), and the developed imaging workflow for MRI-guided IVC filter intervention (**C**). The corresponding workflow for SNL-assisted IVC filter retrieval is provided in ESM-6. ¹ Femoral access was used in the present study; the vascular access route depends on the filter configuration and retrieval hook orientation

A dedicated MRI-adapted workflow for catheter-based IVC filter implantation and retrieval was established (Fig. 4).

### Stage II: in vitro phantom-based IVC filter interventions

**Fig. 5:**
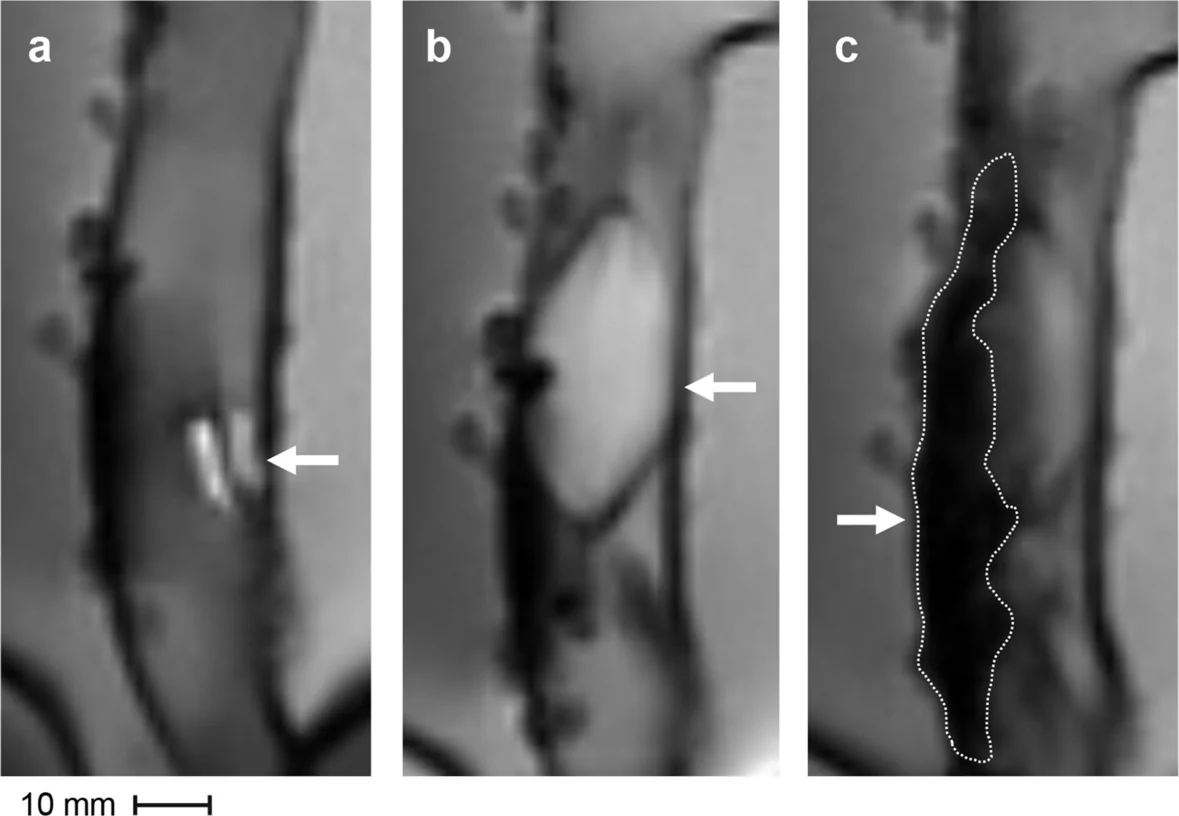
MRI visualization of a semi-active marker–equipped IVC filter delivery system and a resonant inferior vena cava (IVC) filter in the vessel phantom. **a** Semi-active marker–equipped IVC filter delivery system (white arrow) within the inferior vena cava of the vessel phantom. **b** Deployed resonant IVC filter (white arrow) within the inferior vena cava of the vessel phantom demonstrating localized signal enhancement. **c** Deployment catheter–induced in vitro thrombus flushed through the inferior vena cava phantom and captured by the deployed IVC filter (white arrow). The white dotted outline indicates the hypointense area associated with the captured thrombotic material, including material extending cranially from the filter. MRI was performed using a customized TrueFISP sequence with the following parameters: TR = 394.12 ms, TE = 2.04 ms, ST = 5 mm, FA = 40°, BW = 560 Hz/px, matrix = 208×208, pixel size = 1.44×1.44 mm^2^, FoV = 300×300 mm^2^, as summarized in Table 1. The corresponding cine loop is provided in ESM-8

The vessel phantom enabled MRI-guided positioning of the semi-active delivery system and deployment of the resonant IVC filter with continuous visualization during advancement (Fig. 5a). After deployment, the resonant IVC filter generated localized signal enhancement within the vessel lumen (Fig. 5b). Quantitative line-profile analysis confirmed resonant signal enhancement of both the semi-active marker and the deployed IVC filter according to the predefined threshold criterion (*I*_normalized_ > 1.3; see ESM-7). In addition, an in vitro thrombus flushed through the phantom was successfully captured by the deployed IVC filter (Fig. 5c).

### Stage III: in vivo MRI testing

Initial IVC filter implantation was successful on the first attempt in all five animals (5/5). In three animals, the deployed filters were successfully retrieved using the SNL (3/3) and subsequently successfully re-implanted (3/3), resulting in eight successful MRI-guided filter implantations in total (8/8). Retrieval was not attempted in the first two experiments because they were dedicated to sequence and workflow optimization. No structural failure of the MR-resonant IVC filters occurred during in vivo delivery or retrieval.

**Fig. 6:**
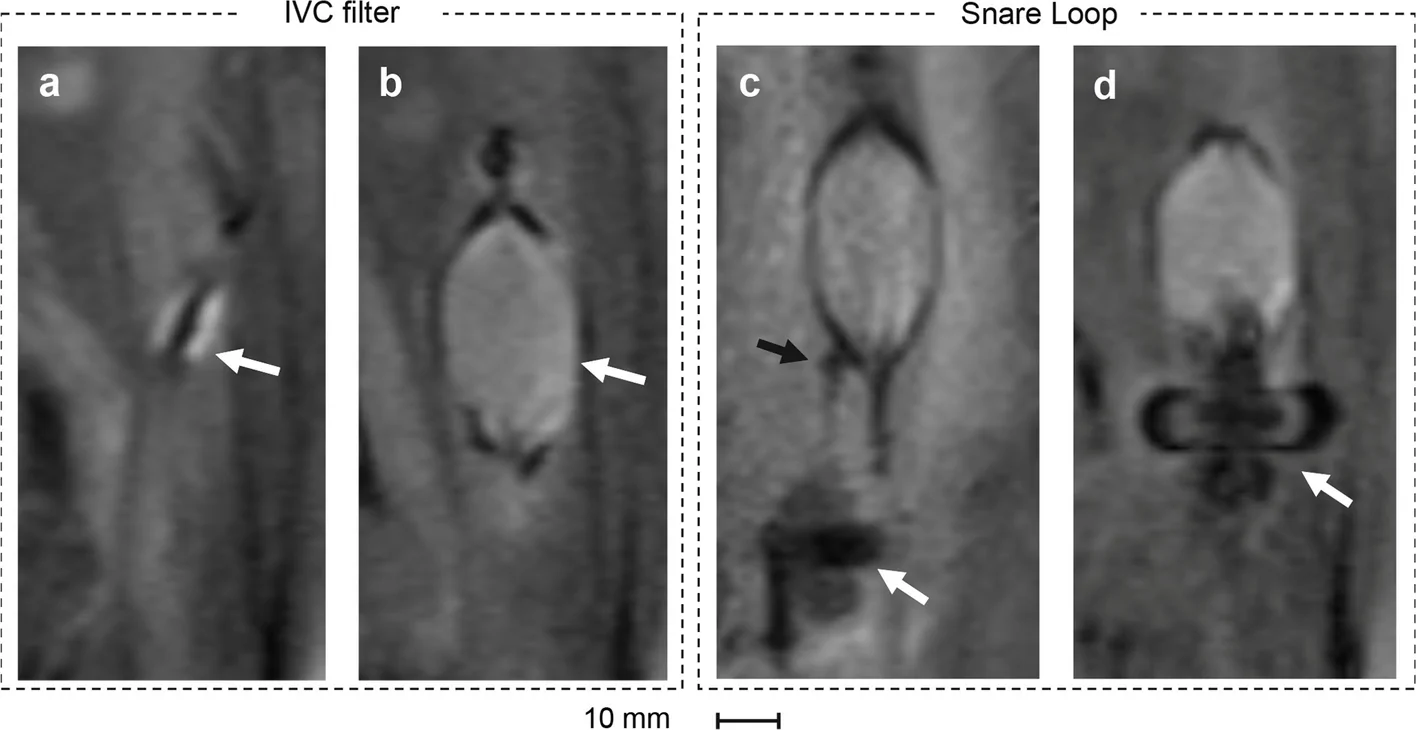
In vivo MRI-guided implantation and snare loop (SNL)-mediated retrieval of resonant inferior vena cava (IVC) filter in a porcine model. **a** Semi-active marker–equipped delivery system (white arrow) enabling continuous visualization during advancement within the inferior vena cava. **b** Deployed resonant IVC filter (white arrow) demonstrating localized signal enhancement and reliable visualization within the caval lumen under physiological conditions. **c** Sagittal-plane MRI during positioning of the SNL at the caudally oriented IVC filter hook during MRI-guided retrieval. The black arrow indicates the SNL, and the white arrow indicates the passively marked delivery catheter. **d** Coronal-plane MRI during positioning of the SNL at the caudally oriented filter hook. The white arrow indicates the passively marked delivery catheter. MRI was performed using a customized TrueFISP sequence with the following parameters: TR = 283.60 ms, TE = 1.36 ms, ST = 4.5 mm, FA = 33°, BW = 850 Hz/px, matrix = 256×256, pixel size = 1.23×1.23 mm^2^, FoV = 316×316 mm^2^, as summarized in Table 1. Corresponding cine loops are provided in ESM-10.1 and ESM-10.2

The semi-active marker-equipped delivery system enabled continuous visualization during advancement within the inferior vena cava (Fig. 6a). After deployment, the resonant IVC filters generated localized signal enhancement within the caval lumen (Fig. 6b). Quantitative line-profile analysis confirmed resonant signal enhancement of all deployed IVC filters according to the predefined threshold criterion (normalized intensity > 1.3; see ESM-9). No resonant signal enhancement of the SNL itself was observed in vivo. Positioning of the SNL at the caudally oriented filter hook (Fig. 6c) was therefore performed using a catheter equipped with passive iron-oxide markers (Fig. 6d). Mean IVC filter implantation and retrieval times were 8.3 ± 4.8 min and 13.1 ± 11.1 min, respectively (mean ± SD).

## Discussion

Initial phantom experiments characterized the MR-resonant signal behavior of the developed IVC filter and snare loop at 3 T. Unlike previous MRI-guided IVC filter studies performed predominantly at 1.5 T [10–12], the present work demonstrates feasibility at 3 T, where increased SNR and stronger resonant amplification may improve device conspicuity but may also require more careful optimization of sequence parameters and RF-related device behavior [19]. MRI-based visualization of the filter hook may facilitate retrieval in cases complicated by fibrin deposition or thrombus formation [11, 12]. These visibility experiments provided the basis for subsequent workflow development and MRI-guided intervention testing. The IVC filter demonstrated stable resonant signal behavior across clinically relevant orientations. The highest signal enhancement observed at a flip angle of 10° likely reflects local RF field amplification by the resonant LC circuit, resulting in increased effective flip angle at the device location, as previously reported [12, 14, 20]. In contrast, the SNL showed orientation-dependent signal amplification, with reduced enhancement in the clinically relevant retrieval orientation. Nevertheless, signal visibility within the loop interior remained sufficient for reliable positioning and engagement of the filter hook under MRI guidance, although further optimization of the resonant loop geometry will be required.

The vessel phantom provided a translational platform between bench-top characterization and in vivo testing, enabling controlled assessment of device visibility, positioning, and deployment while supporting implementation of MRI-guided workflows in accordance with the 3R principle [21]. Phantom-based MRI evaluation of the finalized SNL configuration was not feasible before the in vivo experiments. However, bench-top testing confirmed functional retrieval handling prior to animal studies. Together, these experiments facilitated adaptation of imaging strategies and procedural workflow elements before translation to the porcine model. Imaging strategies required progressive adaptation across study stages. Static high-resolution acquisitions were suitable for characterization of resonant signal behavior in the saline phantom, whereas phantom and in vivo interventions required increasing emphasis on temporal resolution and device tracking. This trade-off between spatial resolution, signal conspicuity, and real-time imaging performance represents a central challenge of MRI-guided endovascular procedures [1, 2]. Previous MRI-guided IVC filter studies primarily demonstrated technical feasibility, whereas structured integration of imaging tasks into the procedural workflow has received less attention [10–12]. The present workflow linked device navigation, deployment, and retrieval to dedicated MRI visualization strategies, thereby extending previous feasibility-focused approaches.

All resonant inferior vena cava filters were successfully implanted under MRI guidance in the porcine model, and SNL-assisted retrieval followed by re-implantation was achieved in all retrieval attempts. During MRI-guided interventions, the semi-active delivery markers and resonant IVC filter provided sufficient visualization for reliable navigation, positioning, and deployment under physiological conditions. As in other MRI-guided endovascular procedures, device visibility depended on slice orientation and imaging plane selection during real-time guidance [1, 2].

The presented design demonstrates the feasibility of integrating electrically separated resonant elements into a retrievable nitinol-based IVC filter while maintaining mechanical stability during catheter-based delivery and retrieval. Successful retrieval and re-implantation without structural failure further confirmed in vivo mechanical integrity of the resonant filter design. In the present configuration, the retrieval hook was oriented caudally to enable femoral retrieval. Alternative configurations with a cranial or potentially double-sided hook design may allow retrieval via different vascular access routes. Previous studies demonstrated MRI-guided placement and experimental retrieval approaches using conventional off-the-shelf filters [10–12]. In contrast, the present study demonstrates MRI-guided implantation and SNL-assisted retrieval of a dedicated MR-resonant retrievable IVC filter within a structured MRI-guided workflow at 3 T. MRI-guided IVC filter implantation may be particularly relevant when radiation or iodinated contrast media are contraindicated. Ultrasound-guided bedside placement represents alternative radiation-free approaches, particularly in critically ill patients [22, 23]. MRI guidance therefore currently represents a specialized investigational approach.

Several limitations of the present multistage preclinical feasibility study should be considered. The study was limited to phantom experiments and acute porcine in vivo evaluation. Despite general anatomical similarities, species-specific differences exist between the porcine caudal vena cava and human IVC, particularly in the hepatic region [24, 25]. Future studies should assess retrieval performance after long-term implantation, including conditions associated with fibrin deposition and endothelial incorporation at the filter hook that may affect retrieval under clinical conditions [9]. Although initial RF-induced heating experiments were performed, comprehensive safety evaluation under clinically relevant imaging conditions remains necessary. Furthermore, device performance was evaluated at 3 T, whereas previous MRI-guided IVC filter approaches were predominantly performed at 1.5 T [10, 12]. The devices could also be used at 1.5 T, but resonant signal amplification would require retuning of the integrated capacitance to the corresponding Larmor frequency. Although functional positioning of the SNL during retrieval was feasible under MRI guidance, further optimization of the resonant loop geometry may improve device conspicuity. The structured multistage evaluation strategy presented here may provide a useful framework for development and assessment of other MRI-guided endovascular devices and interventions, consistent with previous applications in the evaluation of MRI-guided endomyocardial biopsy forceps and MR-compatible stenting approaches.

## Conclusion

This study demonstrates the feasibility of MRI-guided implantation and SNL-assisted retrieval of a dedicated MR-resonant IVC filter at 3 T. Phantom and in vivo experiments confirmed reliable device visualization, procedural guidance, and mechanical integrity under physiological conditions. The presented structured multistage preclinical study design represents a further step toward clinically applicable MRI-guided placement and retrieval of IVC filters. Beyond device evaluation, this framework enabled progressive translation from bench-top characterization to phantom and in vivo testing while supporting implementation of the 3R principles. Such evaluation strategies may facilitate future development, optimization, and training of MRI-guided endovascular interventions.

## Supplementary Information

Additional file 1: Experimental setup for orientation-dependent MR signal characterization of the resonant IVC filter and snare loop SNL.

Additional file 2: Image-based line profile analysis and threshold-based resonant signal analysis.

Additional file 3. In vitro phantom model and procedure for MRI-guided IVC filter implantation.

Additional file 4: Animal study design details & ARRIVE Statements.

Additional file 5: MR visibility of resonant IVC filter and snare loop.

Additional file 6. Flow diagram of SNL-assisted IVC filter retrieval.

Additional file 7. Signal profiles: IVC filter inside in vitro vessel phantom.

Additional file 8. Cine loop of resonant IVC filter implantation in the vessel phantom.

Additional file 9. Signal profiles: IVC filter inside porcine model. 

Additional file 10. Cine loops of in vivo IVC filter implantation and snare loop–mediated retrieval.

## Data Availability

The datasets generated and/or analyzed during the current study are available from the corresponding author on reasonable request, subject to institutional and contractual restrictions.

## Abbreviations

3R: Replacement, reduction, and refinement
ASTM: American Society for Testing and Materials
bSSFP: Balanced steady-state free precession
IVC: Inferior vena cava
LC: Inductor–capacitor
RF: Radiofrequency
SNL: Snare loop
SNR: Signal-to-noise ratio
T: Tesla
