# Supplementary material for: Multistage preclinical evaluation of a retrievable 3 T MR-resonant inferior vena cava filter and snare loop for MRI-guided interventions
Source: CVIR Endovasc. 2026 Sep 14;9:118. doi: 10.1186/s42155-026-00770-z (PMC13575070; doi:10.1186/s42155-026-00770-z)
Supplement: Supplementary file 1 [file 42155_2026_770_MOESM1_ESM.docx]

## ESM-1: Experimental setup for orientation-dependent MR signal characterization of the resonant IVC filter and snare loop SNL

For initial visibility assessment, all devices were positioned in a polymer-based test box (29 × 22 × 19 cm) filled with physiological saline solution (0.9% NaCl). Static bSSFP (TrueFISP) acquisitions were used to characterize resonant signal behavior and overall image appearance. Experimental setup for orientation-dependent MR signal characterization of the 3T resonant IVC filter can be seen in Figure ESM1.1; Orientation adjustment of the IVC filter within the rotation holder can be seen in Figure ESM-1.2. The experimental setup for MR signal characterization of the SNL is shown in Figure ESM-1.3.

**Figure ESM-1.1.** Experimental setup for orientation-dependent MR signal characterization of the 3T resonant IVC filter. The mounting allowed reproducible positioning across measurements without removing and repositioning the device between sequences, facilitating efficient data acquisition and consistent placement for subsequent quantitative analysis. The setup allowed controlled rotation of the IVC filter around its longitudinal axis at predefined angular steps (22.5°, 45°, 67.5°, and 90°) for systematic evaluation of orientation-dependent MR-resonant behavior.

(A) Photograph of the experimental setup showing the IVC filter mounted in a custom-designed 3D-printed holder. The illustrated example corresponds to the 13 mm filter variant. The holder allowed rotation of the device parallel to the main magnetic field (B₀).

(B) Schematic illustration of the rotation mechanism.

**Figure ESM-1.2.** Orientation adjustment of the IVC filter within the rotation holder.

(A) Photograph of the IVC filter struts in the initial reference position (0°).

(B) Photograph after rotation of the filter by 22.5° around its longitudinal axis, illustrating the controlled adjustment of the strut orientation used for orientation-dependent MR signal measurements.

**Figure ESM-1.3.** Experimental setup for orientation-dependent MR signal characterization of the resonant snare loop (SNL) at 3T.

(A) SNL positioned parallel to the main magnetic field (B₀).

(B) Same setup after 90° rotation of the SNL, resulting in an orientation orthogonal to B₀.
