# Supplementary material for: Multistage preclinical evaluation of a retrievable 3 T MR-resonant inferior vena cava filter and snare loop for MRI-guided interventions
Source: CVIR Endovasc. 2026 Sep 14;9:118. doi: 10.1186/s42155-026-00770-z (PMC13575070; doi:10.1186/s42155-026-00770-z)
Supplement: Supplementary file 2 [file 42155_2026_770_MOESM2_ESM.docx]

## ESM-2: Image-based line profile analysis and threshold-based resonant signal analysis

All image analysis was performed using a custom Python-based algorithm developed in PyCharm (JetBrains, Prague, Czech Republic). Single-slice DICOM images were imported and converted to quantitative pixel intensities by applying the DICOM Rescale Slope and Rescale Intercept attributes to the raw pixel values. A user-defined straight sampling line was placed either horizontally or vertically across the region of interest. To improve robustness against local pixel noise, the sampling line was evaluated as a 5-pixel-wide band (±2 pixels around the drawn center line). Intensities along the line were sampled at 500 equidistant positions using bilinear interpolation and averaged across the band, yielding a one-dimensional signal profile (Figure ESM-2.1). A reference region representing the NaCl solution was defined by manually drawing a circular region of interest (ROI), which was automatically mirrored to the opposite end of the sampling line to obtain two reference ROIs. The NaCl reference intensity was calculated as the median of all sampled profile points within the combined NaCl ROIs, providing robustness against isolated outlier pixels.

**Figure ESM-2.1.** Tool for line-profile extraction and reference signal definition.

(A) Example MR image of the IVC filter loaded into the custom Python-based analysis interface.

(B) Placement of the sampling line across the region of interest. The center line (bright yellow) defines the profile direction, while a 5-pixel-wide evaluation band (dark yellow; ±2 pixels around the center line) was used for noise-robust signal extraction.

(C) Manual definition of a circular NaCl reference region of interest (ROI) (solid green line), which was automatically mirrored to the opposite end of the sampling line (dashed green line) to obtain two reference ROIs for calculation of the NaCl reference signal intensity

Artifact-related resonant signal enhancement along the profile was identified using a fixed relative threshold criterion: In accordance with ASTM F2119 and consistent with previously published implementations of the standard, intensity-enhanced artifact regions were identified using a relative threshold criterion. Any profile sample with an intensity exceeding 130% of the NaCl reference median was classified as an artifact-related signal enhancement [1,2]. For visual quality control, the software displayed the full signal profile together with NaCl reference samples, threshold-classified artifact samples, and horizontal lines indicating the NaCl median and applied threshold (see Figure ESM-2.2). For downstream analysis, the complete profile coordinates and intensities, NaCl values, artifact values, threshold parameters, and relevant DICOM sequence metadata were exported to comma-separated value (CSV) files. Quantitative evaluation was based on normalized signal intensity ratios. For each acquisition, the signal profile was normalized to the NaCl reference by dividing all sampled intensities by the median NaCl intensity (I _normalized_ = I _sample_ / I _NaCl median_). This approach ensured robustness against isolated intensity outliers. For the IVC filter experiments, combined intensity plots were generated to visualize signal behavior under varying flip angle and device rotation configuration.

**Figure ESM-2.2.** Example signal profile and threshold-based classification of resonant signal enhancement for resonant IVC filter.

Grayscale intensity values extracted along the sampling line are shown as a one-dimensional signal profile. The NaCl reference median intensity, defined from the higher median value of the two mirrored reference regions, is indicated by a horizontal blue line. The applied relative threshold (130% of the NaCl reference median) is shown as a second horizontal line. Profile samples exceeding this threshold are highlighted in light blue and classified as artifact-related signal enhancement.

Due to the different device orientation, a vertically aligned sampling line was used for snare loop analysis, as illustrated in Figure ESM-2.3 to Figure ESM-2.5.

**Figure ESM-2.3.** GUI-based workflow for line-profile extraction in the snare loop analysis.

(A) Example MR image of the snare loop loaded into the custom analysis interface.

(B) Placement of a vertically oriented sampling line (bright yellow center line) with a 5-pixel-wide evaluation band (dark yellow; ±2 pixels around the center line) adapted to the device orientation.

(C) Definition of the NaCl reference region of interest (ROI) (solid green line), which was automatically mirrored to the opposite end of the sampling line (dashed green line) to obtain two reference ROIs for calculation of the NaCl reference signal intensity.

**Figure ESM-2.4.** Example signal profile and threshold-based classification of resonant signal enhancement for the resonant snare loop.

Grayscale intensity values extracted along the sampling line are shown as a one-dimensional signal profile. The NaCl reference median (blue line) and the applied relative threshold (130%) are indicated. Samples exceeding the threshold (light blue) were classified as artifact-related signal enhancement.

**Sources for ESM-2**

1. Heinrich A, Teichgräber UK, Güttler FV. Measurement of susceptibility artifacts with histogram-based reference value on magnetic resonance images according to standard ASTM F2119. Biomedical Engineering / Biomedizinische Technik [Internet]. 2015 [cited 2023 Aug 3];60. https://doi.org/10.1515/bmt-2014-0184

2. Heinrich A, Güttler F. Software-based automated measurement of susceptibility artifacts on magnetic resonance images. 2011.
