# Supplementary material for: Multistage preclinical evaluation of a retrievable 3 T MR-resonant inferior vena cava filter and snare loop for MRI-guided interventions
Source: CVIR Endovasc. 2026 Sep 14;9:118. doi: 10.1186/s42155-026-00770-z (PMC13575070; doi:10.1186/s42155-026-00770-z)
Supplement: Supplementary file 3 [file 42155_2026_770_MOESM3_ESM.docx]

## ESM-3.1: Details on in vitro phantom

The polymer-based in vitro phantom for simulation of MRI-guided IVC filter implantation was developed with Phacon GmbH (Leipzig, Germany). Blood flow (5 L min^-1^) was simulated using a physiological saline solution mixed with 30% glycerol (WHC GmbH, Hilgertshausen-Tandern, Germany) and a pulsatile flow pump (MultiFlow centrifugal pump, GAMPT mbH, Merseburg, Germany). Femoral access for interventional devices such as guidewires and catheters were realized by puncturing silicone tubes (Deutsch & Neumann GmbH, Hennigsdorf, Germany) at the proximal femoral section of the polymer-based vascular phantom (outside the housing) with lumen diameters of 16 mm (wall thickness: 2 mm).

## ESM-3.2: Details on phantom-based IVC filter implantation

MRI-guided IVC filter implantation was simulated using the vascular phantom to assess procedural feasibility with MR Compatible devices. After initial plane planning and definition of the target region for filter deployment, a 0.035" (0.89 mm), 260 cm hydrophilic-coated MR Conditional guidewire (REF 39012526, Epflex Feinwerktechnik GmbH, Dettingen an der Erms, Germany) was advanced into the inferior vena cava. Subsequently, the IVC filter delivery system (OD 5.2 mm, approximately 16 Fr) mounted on a long dilator was introduced over the guidewire and positioned using the MR-resonant marker. The dilator and guidewire were then withdrawn, and the IVC filter, mounted on a polymer holder catheter, was advanced through the delivery system until reaching the predefined marker position. After correct positioning, the IVC filter was deployed by pullback of the delivery system and advancement of a dedicated fiberglass pusher rod. For simulated right femoral vein access outside the MRI suite under ultrasound (US) guidance, an 18 G MR cannula (Epflex Feinwerktechnik GmbH), a short guidewire (REF 46012082, Epflex Feinwerktechnik GmbH), and an 18 Fr non-braided Performer® introducer (Cook Medical, Bloomington, IN, USA) were used.
