# Supplementary material for: Multistage preclinical evaluation of a retrievable 3 T MR-resonant inferior vena cava filter and snare loop for MRI-guided interventions
Source: CVIR Endovasc. 2026 Sep 14;9:118. doi: 10.1186/s42155-026-00770-z (PMC13575070; doi:10.1186/s42155-026-00770-z)
Supplement: Supplementary file 4 [file 42155_2026_770_MOESM4_ESM.docx]

**ESM-4 Animal study design details & ARRIVE Statements**

**ESM-4.1 Detailed in vivo MRI-guided implantation and retrieval procedure**

Five female pigs (*Sus scrofa domesticus*) aged 12$-$16 weeks and weighing 34$-$58 kg were included in the MRI-guided caval interventions. All in vivo procedures were performed within the framework of a larger ethically approved animal study evaluating the feasibility of MRI-guided cardiovascular interventions and an interventional MRI setup [1,2]. The study design followed the ARRIVE guidelines and was exploratory in nature with the primary aim of assessing procedural feasibility; therefore, no control group was included. Animal preparation, including anesthesia, physiological monitoring, vascular access, positioning, and in-room handling, followed a previously established protocol [3], which provides detailed descriptions of sedation, intubation, anesthesia maintenance, and monitoring procedures are provided. Vascular access was obtained by ultrasound-guided percutaneous puncture of the right femoral vein using the Seldinger technique outside the MRI suite. Subsequently, the animals were transferred to the MRI system and positioned supine on the scanner table. All IVC filters were deployed in an infrarenal position and subsequently retrieved via the same femoral access route. All interventions were conducted on a 3.0 T PET/MRI system (Biograph mMR, Siemens Healthineers) using the established MRI-guided workflow and the MR-compatible devices described above.

After deployment, the IVC filter was retrieved under MR guidance using an SNL-based sheath retrieval technique in combination with a passively marked retrieval catheter. Following MR visualization, the IVC filter was retrieved using the SNL system. The SNL, integrated into its dedicated delivery catheter (OD 2.3 mm, approximately 7 Fr), was advanced through a passively (FeO_2_) marked retrieval catheter (OD 3.8 mm, approximately 11 Fr), positioned around the caudal retrieval hook, tightened, and the filter was subsequently withdrawn into the catheter under MR guidance.

**Figure ESM-4.1. Interventionist positioning and procedural setup during MRI-guided caval intervention.** The interventionist is indicated by a white arrow in both panels. (a) The interventionist was positioned on the animal’s right side close to the scanner bore, with the arms reaching into the bore for device manipulation through the femoral sheath. (b) During real-time MRI guidance, the interventionist maintained direct view of the projected MR images. Communication with the MRI technologists in the control room was enabled via headset and microphone.

**ESM 4.2 ARRIVE Statements**

The animal experiments were performed as part of an exploratory preclinical program on MRI-guided cardiovascular interventions. Details of the overall study setting have been reported previously [3]. The present analysis focuses on the subgroup of animals that underwent MRI-guided caval intervention. All procedures were approved by the competent animal welfare authority, Landesdirektion Sachsen, and were conducted in accordance with the German Animal Welfare Act and relevant European legislation, including Directive 2010/63/EU [2]. The experimental unit was the individual animal. Because the study was designed as a feasibility investigation, the sample size was based on procedural and technical feasibility considerations rather than on a formal a priori power calculation. The overall exploratory study included ten healthy female domestic pigs (Sus scrofa domesticus), aged 12–16 weeks and weighing 34–58 kg. Five of these animals underwent the MRI-guided caval intervention investigated in the present work. Assignment to this intervention was determined by procedural workflow and the planned sequence of technical test procedures within the broader study. No animal assigned to MRI-guided caval intervention was excluded from the analysis. Eligible animals were female, clinically healthy, and considered suitable for general anesthesia and catheter-based intervention. Animals not meeting these criteria would have been excluded. Randomization and blinding were not applied because of the technical and procedural nature of the feasibility study. The assessed outcomes comprised procedural feasibility, MRI visibility of the devices, successful stent deployment, and intervention duration. Animals were euthanized at the end of the procedure according to the approved protocol. Humane endpoints and monitoring criteria were defined in the approved animal welfare score sheet.

**Sources for ESM-4**

1. Reich CM, Sattler B, Jochimsen TH, Unger M, Melzer L, Landgraf L, et al. Practical setting and potential applications of interventions guided by PET/MRI. Q J Nucl Med Mol Imaging [Internet]. 2021 [cited 2023 Aug 16];65. https://doi.org/10.23736/S1824-4785.20.03293-8

2. Landesdirektion S. Approval of animal experiment protocol [TVV 54/21]. 2022.

3. Gholami Bajestani D, Reich CM, Birkigt J, Mulik C, Hiller GGR, Busse H, et al. Development and application of a structured preclinical protocol for MRI-guided endomyocardial biopsy: a multimodal imaging approach using a hybrid vessel phantom and porcine in vivo models. Z Med Phys. 2026;S0939-3889(25)00143-6. https://doi.org/10.1016/j.zemedi.2025.11.001
