# Supplementary material for: Multistage preclinical evaluation of a retrievable 3 T MR-resonant inferior vena cava filter and snare loop for MRI-guided interventions
Source: CVIR Endovasc. 2026 Sep 14;9:118. doi: 10.1186/s42155-026-00770-z (PMC13575070; doi:10.1186/s42155-026-00770-z)
Supplement: Supplementary file 5 [file 42155_2026_770_MOESM5_ESM.docx]

## ESM-5: MR visibility of resonant IVC filter and snare loop

**Figure ESM-5.1.** Representative MR images corresponding to the parameter combinations yielding the highest and lowest calculated normalized signal intensities of the resonant IVC filter (white arrows) for both diameter configurations.

(A) MR image corresponding to the maximum normalized signal intensity for the 13 mm filter (flip angle 10°, rotation 22.5°).

(B) MR image corresponding to the minimum normalized signal intensity for the 13 mm filter (flip angle 40°, rotation 90°).

(C) Transverse gradient-echo image of the 13 mm filter illustrating the cross-section of the filter structure.

(D) MR image corresponding to the maximum normalized signal intensity for the 20 mm filter (flip angle 10°, rotation 22.5°).

(E) MR image corresponding to the minimum normalized signal intensity for the 20 mm filter (flip angle 40°, rotation 90°).

(F) Transverse gradient-echo image of the 20 mm filter illustrating the cross-section of the filter structure.

3 T Siemens Biograph mMR imaging parameters were as follows:

A, D: Customized TrueFISP: TR = 428.83 ms, TE = 2.22 ms, ST = 3 mm, FA = 10°, BW = 560 Hz/px, matrix = 208 × 208, pixel size = 1.50 × 1.50 mm², FoV = 310 × 310 mm².

B, E: Customized TrueFISP: TR = 428.83 ms, TE = 2.22 ms, ST = 3 mm, FA = 40°, BW = 560 Hz/px, matrix = 208 × 208, pixel size = 1.50 × 1.50 mm², FoV = 310 × 310 mm².

C, F: Gradient-echo sequence: TR = 270 ms, TE = 3.54 ms, ST = 2.5 mm, FA = 20°, BW = 605 Hz/px, matrix = 192 × 120, pixel size = 1.11 × 1.11 mm², FoV = 133 × 214 mm².

**Figure ESM-5.2.** Representative MR images illustrating orientation-dependent signal behavior of the snare loop (white arrows) relative to the imaging plane at a flip angle of 40°.

(A) Loop plane-oriented parallel to the transverse imaging plane, corresponding to the expected in vivo configuration during inferior vena cava filter retrieval. No signal enhancement exceeding the predefined threshold (normalized intensity > 1.3) was observed. Signal within the loop interior remained preserved without signal extinction.

(B) Loop plane-oriented perpendicular to the transverse imaging plane (sagittal orientation), resulting in orientation-dependent resonant signal enhancement exceeding the threshold criterion. Signal maxima were observed along the inner margins of the loop structure with a gradual decrease toward a central plateau region within the loop interior.

Customized TrueFISP: TR = 428.83 ms, TE = 2.04 ms, ST = 10 mm, FA = 40°, BW = 560 Hz/px, matrix = 208 × 208, pixel size = 1.44 × 1.44 mm², FoV = 300 × 300 mm².

**Figure ESM-5.3.** Signal profile and threshold-based classification of resonant signal enhancement for snare loop. Snare loop plane oriented parallel to transverse imaging plane (corresponding to the expected application orientation during filter retrieval in the inferior vena cava).

Grayscale intensity values extracted along the sampling line are shown as a one-dimensional signal profile. The NaCl reference median intensity, defined from the higher median value of the two mirrored reference regions, is indicated by a horizontal green line. No signal enhancement exceeding the predefined threshold (I _normalized_ > 1.3) was observed.

**Figure ESM-5.4.** Signal profile and threshold-based classification of resonant signal enhancement for snare loop. Snare loop plane oriented sagittal to transverse imaging plane.

Grayscale intensity values extracted along the sampling line are shown as a one-dimensional signal profile. The NaCl reference median intensity, defined from the higher median value of the two mirrored reference regions, is indicated by a horizontal blue line. The applied relative threshold (130% of the NaCl reference median) is shown as a second horizontal line. Profile samples exceeding this threshold are highlighted in light blue and classified as artifact-related signal enhancement.
