# Supplementary material for: Multistage preclinical evaluation of a retrievable 3 T MR-resonant inferior vena cava filter and snare loop for MRI-guided interventions
Source: CVIR Endovasc. 2026 Sep 14;9:118. doi: 10.1186/s42155-026-00770-z (PMC13575070; doi:10.1186/s42155-026-00770-z)
Supplement: Supplementary file 6 [file 42155_2026_770_MOESM6_ESM.docx]

**ESM-6 SNL-assisted filter retrieval**

Figure ESM-6.1. Flow diagram of SNL-assisted IVC filter retrieval (A), including the standard imaging workflow for fluoroscopy guidance (B) and the developed imaging workflow for MRI guidance (C).

¹ Femoral access was used in the present study; the vascular access route depends on the filter configuration and retrieval hook orientation.
