# Supplementary material for: Multistage preclinical evaluation of a retrievable 3 T MR-resonant inferior vena cava filter and snare loop for MRI-guided interventions
Source: CVIR Endovasc. 2026 Sep 14;9:118. doi: 10.1186/s42155-026-00770-z (PMC13575070; doi:10.1186/s42155-026-00770-z)
Supplement: Supplementary file 7 [file 42155_2026_770_MOESM7_ESM.docx]

## ESM-7: Signal profiles: IVC filter inside in vitro vessel phantom

**Figure ESM-7.1.** Signal profile and threshold-based classification of resonant signal enhancement for IVC filter inside vessel phantom.

Grayscale intensity values extracted along the sampling line are shown as a one-dimensional signal profile. The NaCl reference median intensity, defined from the higher median value of the two mirrored reference regions, is indicated by a horizontal green line. The applied relative threshold (130% of the NaCl reference median) is shown as a second horizontal line. Profile samples exceeding this threshold are highlighted in light blue and classified as artifact-related signal enhancement.

**Figure ESM-7.2.** Signal profile and threshold-based classification of resonant signal enhancement for Semiactive marker–equipped IVC filter delivery system inside vessel phantom.

Grayscale intensity values extracted along the sampling line are shown as a one-dimensional signal profile. The NaCl reference median intensity, defined from the higher median value of the two mirrored reference regions, is indicated by a horizontal green line. The applied relative threshold (130% of the NaCl reference median) is shown as a second horizontal line. Profile samples exceeding this threshold are highlighted in light blue and classified as artifact-related signal enhancement.
