# Supplementary material for: Multistage preclinical evaluation of a retrievable 3 T MR-resonant inferior vena cava filter and snare loop for MRI-guided interventions
Source: CVIR Endovasc. 2026 Sep 14;9:118. doi: 10.1186/s42155-026-00770-z (PMC13575070; doi:10.1186/s42155-026-00770-z)
Supplement: Supplementary file 8 [file 42155_2026_770_MOESM9_ESM.docx]

## ESM-9: Signal profiles: IVC filter inside porcine model

**Figure ESM-9.1.** Normalized MR signal intensity maxima of the resonant IVC filters (n = 5) in five in vivo experiments.

Signal maxima exceeding the predefined threshold (normalized intensity > 1.3) are indicated by black downward-pointing triangles. Results are shown separately for each implanted IVC filter. For the in vivo evaluation, the surrounding tissue was used as the reference for median intensity calculation. Intensities were normalized to the surrounding tissue reference signal (I _normalized_ = I _IVC filter_ / I _surrounding tissue median_).
